# Remote continuous monitoring with wireless wearable sensors in clinical practice, nurses perspectives on factors affecting implementation: a qualitative study

**DOI:** 10.1186/s12912-022-00832-2

**Published:** 2022-03-07

**Authors:** Laura Kooij, Guido M. Peters, Carine J. M. Doggen, Wim H. van Harten

**Affiliations:** 1 Rijnstate, Arnhem, the Netherlands; 2 Department of Health Technology and Services Research, Techmed Centre, University of Twente, Drienerlolaan 5, Enschede, the Netherlands; 3 Rijnstate, Clinical Research Center, Arnhem, the Netherlands; 4 Division of Psychosocial Research and Epidemiology, The Netherlands Cancer Institute, Amsterdam, the Netherlands

**Keywords:** Continuous monitoring, Wireless technology, Nurses

## Abstract

**Background:**

Continuous monitoring using wireless wearable sensors is a promising solution for use in clinical practice and in the home setting. It is important to involve nurses to ensure successful implementation. This paper aims to provide an overview of 1) factors affecting implementation of continuous monitoring using wireless wearable sensors by evaluating nurses’ experiences with its use on the nursing ward, and 2) nurses’ expectations for use in the home setting.

**Methods:**

Semi-structured interviews were conducted with 16 nurses from three teaching hospitals in the Netherlands, covering constructs from the Consolidated Framework for Implementation Research (CFIR). A deductive approach of directed content analysis was applied. One additional factor was added using the Unified Theory for Acceptance of Technology (UTAUT). The quotes and domains were rated on valence (positive, neutral, negative) and strength (strong: − 2, + 2, neutral 0, and weak: − 1, + 1).

**Results:**

Data was collected on 27 CFIR constructs and 1 UTAUT construct. In the experience of at least 8 nurses, five constructs had a strong positive influence on implementation on the nursing ward, including relative advantage (e.g., early detection of deterioration), patient needs and resources (e.g. feeling safe), networks and communications (e.g. execute tasks together), personal attributes (e.g. experience with intervention), and implementation leaders (e.g., project leader). Five constructs had a strong negative influence: evidence strength and quality (e.g. lack of evidence from practical experience), complexity (e.g. number of process steps), design quality and packaging (e.g., bad sensor quality), compatibility (e.g, change in work) and facilitating conditions (e.g, Wi-Fi connection). Nurses expected continuous monitoring in the home setting to be hindered by compatibility with work processes and to be facilitated by staff’s access to information. Technical facilitating conditions (e.g. interoperability) were suggested to be beneficial for further development.

**Conclusions:**

This paper provides an overview, of factors influencing implementation of continuous monitoring including relative importance, based on nurses’ experiences with use on nursing wards, and their perspectives for use in the home setting. Implementation of continuous monitoring is affected by a wide range of factors. This overview may be used as a guideline for future implementations.

**Supplementary Information:**

The online version contains supplementary material available at 10.1186/s12912-022-00832-2.

## Background

Patients’ vital signs are monitored during hospitalization to detect clinical deterioration. Vital signs are monitored continuously on Intensive Care Units (ICU), while patients on clinical wards are generally monitored intermittently [1, 2], often every 4 [3], or 6 to 8 h [2]. Several parameters are measured during these routine observations including heart rate, respiratory rate and blood pressure. Nursing staff usually conducts these measurements in person, which can be time consuming [2]. A Modified Early Warning Score (MEWS), a scoring system incorporating all intermittent measurements and other observations, is often used to facilitate detection of clinical deterioration on nursing wards [4].

Continuous monitoring of vital signs using wireless wearable sensors in nursing wards is a promising solution and may lead to earlier detection of deterioration in patients [5], early interventions [6], reduction in length of stay, and fewer ICU days [2]. It may also contribute to patient safety [7], improve patient mobility [7], and reduce workload for nurses [8]. Many different wearable sensors are available [1]. Some provide functionality similar to that of monitors on the Intensive Care Unit. These tend to be cumbersome devices. Other sensors measure a limited number of vital signs, such as heart rate and respiratory rate, but come in more manageable forms, such as adhesive patches that can be attached to a patient’s chest. These are more suitable for monitoring on the nursing ward, and may also be suitable for use in the home setting [9]. The implementation and use of these sensors is likely to affect hospital staff and their work. Therefore, the involvement of nursing staff, who are often responsible for the monitoring of patients, is essential for successful implementation in clinical practice [3, 5].

Implementation of technology such as continuous monitoring in clinical practice is a complex process [10–12], which can be affected by technical, social and organizational factors [12, 13]. It is valuable to engage stakeholders throughout the whole process, including development and evaluation [10]. When stakeholders are not involved in the implementation process, this has been found to be a barrier for implementation [3]. Therefore it is important to obtain their input [14]. Previous studies found that nurses are positive about the possible benefits of continuous monitoring, such as signaling early deterioration, but they also see disadvantages, for example less patient contact [15] and technical issues [16]. Recent evidence on the use of wireless sensors in daily clinical practice is limited [3]. Some positive and negative factors affecting implementation of continuous monitoring with wireless wearable sensors were reported in previous studies [3, 9, 15]. However, systematic information on the relative importance of a broad range of factors affecting implementation, especially from the perspective of nurses, is limited. Also, there is a lack of insight into nurses’ expectations regarding future developments in continuous monitoring using these sensors in the home setting, and how this could change nurses’ roles.

The aim of this paper is to provide an overview of 1) factors affecting implementation of continuous monitoring using wireless wearable sensors by evaluating nurses’ experiences with its use on the clinical ward, and of 2) nurses’ expectations for its use in the home setting.

## Methods

### Sampling procedure and participants

We conducted a qualitative study, with a generic approach [17]. Hospitals in the Netherlands where continuous monitoring with wireless wearable sensors was used, further referred to as continuous monitoring, were selected using purposive sampling. In total, 3 teaching hospitals were included, through the authors’ network. Department heads or managers in the hospitals were approached by e-mail or telephone to invite hospital nurses to participate in the study. The criterion was that the nurses had been involved in continuous monitoring using sensors on the nursing ward. Semi-structured interviews were scheduled individually with the nurses who agreed to participate in the study. No preparation was requested.

### Data collection procedure

The content of the semi-structured interviews was based on the Consolidated Framework for Implementation Research (CFIR). This framework describes constructs organized in 5 domains: 1) Intervention Characteristics, e.g. evidence strength & quality and complexity, 2) Outer Setting, e.g. cosmopolitanism and patient needs & resources, 3) Inner Setting, e.g. compatibility and networks & communication, 4) Characteristics of Individuals, e.g. knowledge & beliefs and other personal attributes, and 5) Process, e.g. champions and reflecting & evaluating [11]. The interview guide can be found in Additional file 1.

The interviews were conducted by the first author (LK). At the start of the interview, all respondents were informed about the purpose of the interview, and verbal consent for audio recording was obtained. Demographic data on sex, age, and work experience were collected at the start of the interview. The 10 interviews in the first hospital were conducted face-to-face in a room on the nursing ward with only the respondent and interviewer present. The other interviews, 3 interviews in two hospitals each, were conducted by telephone due to COVID-19 circumstances. The interviews lasted 31.5 min (range 19–44 min) on average. Data were collected between December 2019 and July 2020. Ethical approval for this study was asked for and waived by the Medical Research Ethics Committee Arnhem-Nijmegen (registration 2019–5489). The study fell outside the remit of the law for Medical Research Involving Human Subjects Act and was approved by the local ethical committee.

### Data analysis

The first author (LK) transcribed all interviews verbatim. Transcripts were anonymized and not returned to the participants. The first two authors (LK, GMP) independently selected text fragments (‘quotes’) and coded all interviews using Atlas.ti version 8. If quotes did not fit in the CFIR framework, the Unified Theory of Acceptance and Use of Technology (UTAUT) was used for other specific technological aspects, in particular facilitating conditions [18]. A deductive approach of directed content analysis [19] was applied. Although we did not develop questions for all CFIR constructs, some topics came up during interviews nonetheless. These topics were coded to the corresponding CFIR construct.

Subsequently, the first two authors (LK, GMP) rated the valence and strength of each quote using CFIR criteria (see Additional file 2). The valence could be positive, negative or neutral. The valence for the total could also be mixed (both positive and negative). The strength indicated whether a construct had a weak (− 1 or + 1), strong (− 2 or + 2), or neutral (0) influence on implementation [20]. The first two authors rated each CFIR construct on valence and strength, and a case memo [21] was written (Additional file 3). Inconsistencies in coding and rating between the two assessors were resolved through discussion. If no agreement was reached, a third assessor (CD) provided adjudication. Saturation of the data was analyzed (post hoc) and confirmed, indicating that more interviews would not be likely to lead to additional new factors. Feedback on the findings was not elicited from participants. For the reporting of this paper, we used the COREQ guidelines (Additional file 4). Descriptive statistics, including mean (standard deviation (SD)), number, and percentages, were analyzed with IBM SPSS V22.0.

## Results

### Study population

In total, 16 interviews were conducted with nurses from three teaching hospitals. Their characteristics are presented in Table 1.

**Table 1:** Characteristics of the 16 participating nurses

Characteristics	Nurses (*N* = 16)
Sex, women, n(%)	16 (100)
Age, mean (SD), range (min-max)	34.1 (11.2), 22–56
Work experience	
0–4 years	3 (19)
5–9 years	4 (25)
10–14 years	4 (25)
> =15 years	5 (31)

### Intervention

All three hospitals used continuous monitoring on the nursing ward, though for different patient groups. Hospital 1 used continuous monitoring for bariatric patients after surgery, Hospital 2 for patients who had undergone heart- or heart valve surgery, for clinically unstable patients, and for vulnerable elderly patients, and Hospital 3 used it for patients with pulmonary, neurological, gastrointestinal, and liver diseases. Two out of three hospitals used the Philips Biosensor [22] to measure heart rate and respiratory rate. The third hospital used a sensor from SensiumPatch [23] to measure heart rate, respiratory rate and temperature. The Philips Biosensor has a battery life of 4 days, while that of the Sensium Patch is 4–5 days.

### Experiences with continuous monitoring on the nursing ward and in the home setting

In total, we selected 1068 quotes covering 27 CFIR constructs and 1 UTAUT construct. A total overview of the rating of all quotes from all respondents can be found in Additional files 5 (nursing ward) and Additional file 6 (home setting). We quantified the findings, and present the most prevailing results below.

On the nursing ward, 19 CFIR constructs and 1 UTAUT construct were identified by at least 8 nurses. Of these, 10 constructs had a positive influence, 5 were mixed, and 5 had a negative influence on implementation of continuous monitoring on the nursing ward. In the home setting, seven constructs were identified by at least 8 nurses, 2 of which were projected to have a positive influence, 2 a negative influence, while 3 were mixed. Constructs that were mentioned by at least 8 (out of 16) nurses are described below and presented in Table 2.

**Table 2:** Continuous monitoring on the nursing ward and expectations for use in the home setting (*N* = 16)

CFIR and UTAUT constructs	Experiences: on the nursing ward		Expectations: use in the home setting	
	Total rating^a^	Total N nurses (no. of quotes^b^)	Total rating^a^	Total N nurses (no. of quotes^b^)
**I. Intervention characteristics**				
Evidence Strength and Quality	−2	14(36)	−1	8(18)
Relative advantage	+ 2	15(61)	+ 2	10(16)
Trialability	Mixed	8(15)	–^c^	–
Complexity	−2	16(100)	NA^d^	NA
Design Quality and Packaging	−2	15(39)	–	–
**II. Outer setting**				
Patient needs & resources	+ 2	10(25)	Mixed	15(44)
**III. Inner setting**				
Networks & Communications	+ 2	15(32)	NA	NA
Tension for change	+ 1	13(18)	NA	NA
Compatibility	−2	13(39)	−2	16(97)
Relative priority	Mixed	16(39)	NA	NA
Goals and Feedback	+ 1	16(20)	NA	NA
Learning Climate	+ 1	16(79)	NA	NA
Available resources	Mixed	16(42)	Mixed	9(14)
Access to information and knowledge	+ 1	16(48)	+ 2	13(21)
**IV. Characteristics of individuals**				
Knowledge and beliefs	Mixed	9(12)	Mixed	12(26)
Other personal attributes	+ 2	12(19)	NA	NA
**V. Process**				
Formally Appointed Internal Implementation Leaders	+ 2	10(26)	–	–
Champions	+ 1	14(34)	–	–
Reflecting and Evaluating	Mixed	15(33)	–	–
**UTAUT**				
Facilitating conditions	−2	8(31)	–	–

^a^A minus sign (−) means a negative influence on implementation. A positive sign (+) means

positive influence on implementation. ‘Mixed’ means both negative and positive influence on implementation

^b^In total, 1068 quotes were selected of which 5 quotes were coded to two constructs

^c^ –: construct was not mentioned by nurses

^d^Not applicable (NA): mentioned by 1–7 nurses

### Intervention characteristics

#### Evidence strength and quality

##### Experience on the nursing ward

This domain refers to respondents’ practical experiences on the nursing ward and perceptions of the available evidence (e.g. from use in practice) for continuous monitoring. Statements about the importance of evidence strength and quality of continuous monitoring on the nursing ward were made by almost all respondents (14/16, 88%), with a strong negative influence on implementation. Respondents referred especially to the lack of available evidence to substantiate the use of continuous monitoring with a limited number of vital signs, e.g. only heart-rate and respiratory rate, in their patient population. Gathered evidence based on practical experiences was also found to be a negative influence, especially because measurements of vital signs by the sensor often did not correspond with measurements by another monitoring device used in daily practice. Technical issues (e.g., system was not working or not reliable) were also mentioned. Despite a negative sentiment, two nurses mentioned positive experiences with regards to early detection of deterioration (see Additional file 5).

*“We need to gain trust in the idea that heart rate and respiratory rate together provides sufficient information to conduct interventions. That is still difficult for me.”*

##### Expectations for continuous monitoring in the home setting

Half of the nurses (8/16, 50%) were not convinced that there is enough available evidence for continuous monitoring in the home setting. This was caused by predominantly negative experiences based on use on the nursing ward. Additionally, they still need to gain trust in the system and the new way of working (see Additional file 6).

*“We are not even close to monitoring patients at home. Even here [on the nursing ward] it has not worked 100% of the time.”*

#### Relative advantage

##### Experience on the nursing ward

A score of advantages for continuous monitoring were mentioned by almost all nurses (15/16, 94%). Advantages included data availability, patient safety, early discharge, higher turnover, higher quality of measurements, and support of the clinical view. Early detection of deterioration (12/16, 75%) as well as time and efficiency (10/16, 63%) were also seen as advantages. They stated the intervention saves them time as it eliminated the need for measuring vital signs manually during routine rounds.

*“You have a continuous view on the patient. I think that is most important, you can detect early deterioration”*

##### *Expectations for continuous monitoring in the home setting*

Nurses (9/16, 56%) foresee a number of advantages for the use of continuous monitoring in the home setting including data availability, early discharge, higher turnover or lower cost, early deterioration, time or efficiency benefits, and patient safety.

*“The advantage is that people don’t need to spend the night here in the hospital. I think this also saves healthcare costs.”*

#### Trialability

##### Experience on the nursing ward

This domain includes statements on the ability to pilot the intervention. On the one hand, conducting a pilot was perceived positively (3/16, 19%), because the pilot made it possible to gain experience with continuous monitoring. On the other hand, it was perceived negatively (3/16, 19%), because the pilot setting led to additional tasks and duplications in registration due to the use of multiple systems.

*“We conducted a pilot on the nursing ward…I think for a certain number of patients. Based on that pilot we wanted to see if it would be meaningful.”*

#### Complexity

##### *Experience on the nursing ward*

Complexity refers to the perceived difficulty of the intervention. All nurses (16/16, 100%) brought up aspects related to the high degree of complexity of the intervention, and overall complexity was seen as a (strong) negative influence on implementation. The negative rating was especially due to the duration of the intervention (13/16, 81%), perceived difficulty (8/16, 50%), and the number of procedural steps (8/16, 50%). The duration of the intervention relates to the additional time involved with using it, for example to attach and activate the sensor.

*“First, we had to open the system, search for the patient in the system. That will already take approximately 5 minutes, so it takes extra time.”*

#### Design quality and packaging

##### Experience on the nursing ward

The design quality and packaging includes statements regarding the quality of the sensor (e.g, flexibility and attachment to the body), the system (e.g. scanning and connection with sensor) and data availability (e.g. gaps in data availability). The majority of the nurses (13/16, 81%) was not satisfied with the quality of the sensor, for example because of detachment of the sensor from the patient’s body. They were also not satisfied with the quality of the system (3/16, 19%), and data availability (3/16, 19%). Positive elements about the quality of the sensor were only mentioned by a small number of nurses (5/16, 31%). Examples of such positive elements include good attachment of the sensor to the body and flexibility of the sensor.

*“Our target population was sweating a lot after surgery, and we noticed the sensor would come off…”*

### Outer setting

#### Patient needs and resources

##### Experience on the nursing ward

This construct includes factors affecting patients as a result of continuous monitoring in the nursing ward. This was seen as having a positive influence on implementation. One third of the nurses (5/16, 31%) perceived that patients on the nursing ward felt safer when they were monitored continuously, and that they were not burdened by the sensor (5/16, 31%). A minority (3/16, 19%) mentioned that the sensor may be inconvenient for some patients, for example due to skin irritation.

*“There were also patients that felt safe: ‘so you monitor my values 24 hours per day. So even if you are not in my room, you monitor me’. That gave patients a feeling of safety.”*

##### Expectations for continuous monitoring in the home setting

The majority of the nurses (10/16, 63%) mentioned that the intervention can be beneficial for patients because they can recover in their own home. Although 31% (5/16) of the nurses expect that continuous monitoring will make patients feel safe at home, the majority (10/16, 63%) think that early discharge with continuous monitoring might also cause patients to feel insecure or anxious. Adequate patient information is considered an important facilitator (2/16, 13%).

*“I think that people will recover better at home. I also think they will sleep better in their own bed, because that is more pleasant.”*

### Inner setting

#### Networks and communication

##### Experience on the nursing ward

This domain includes nurse preferences for and experiences with communication about the implementation of the intervention. Most nurses (10/16, 63%) were positive about executing a task together with a colleague. They perceived this as a facilitating factor to practice the use of the sensor. Nurses were also positive about both formal communication (8/16, 50%), such as planned information meetings, and informal communication (5/16, 31%) with colleagues.

*“During the planned meetings we could get together and share experiences, we also had frequent mail contact but the moments together were the most pleasant.”*

#### Tension for change

##### Experience on the nursing ward

Tension for change encompasses statements on the need to change the current situation of monitoring on the nursing ward, such as the current practice of using MEWS to detect patient deterioration. Although according to 31% (5/16) changing the current situation would be beneficial e.g. the respiratory rate can be measured by a device instead of manually by nurses, 50% (8/16) did not feel the need to change the current situation. They were satisfied with the current monitoring and especially the use of the Modified Early Warning Score (MEWS).

*“These check-ups, the MEWS, are really useful during acute situations. You can really compare with other check-ups or with deteriorating patients, so I am used to working with the MEWS and I think it is quite nice.”*

#### Compatibility

##### Experience on the nursing ward

This domain relates to the extent to which the intervention is compatible with existing work processes and systems [11]. Multiple (sub)categories were distinguished including compatibility with work processes and the use of systems, change in work, and perceived risks. Compatibility with work processes was rated negatively by most nurses (12/16, 75%). This can be explained by increased workload (4/16, 25%). For example, in case of deteriorating vital signs, nurses needed to check the patients and perform extra check-ups. Some sensor limitations were also not compatible with work processes, according to 6 nurses (6/16, 38%). For instance, the sensor could not measure blood pressure. Also, the sensor could not be used for patients with a pacemaker, when diagnostic tools such as a CT scan were used, or while the patient was taking a shower. Almost half of the nurses (7/16, 44%) thought that with continuous monitoring their work would not change and would not be affected, especially because they think their clinical view is still needed in addition to continuous monitoring. Six nurses (6/16, 38%) reported risks of continuous monitoring including a lack of the clinical view (4/16, 25%).

*“So at some point you could see a deviation in a patient, which you couldn't see with your clinical view alone, but to really be sure how the patient was doing you still had to go and take the measurements. So that was an additional task…”*

##### Expectations for continuous monitoring in the home setting

Compatibility was perceived as a negative influence on implementation of continuous monitoring in the home setting. Half of the nurses (8/16, 50%) thinks that continuous monitoring in the home setting will negatively change their work. According to 44% (7/16) of nurses, the intervention will have a negative effect on their relation and contact with patients, because there will be less personal contact due to patients’ shorter stay in the hospital. In total, 50% (8/16) is negative about compatibility with work processes. Nurses expect workload to increase (5/16, 31%) if they have to monitor patients in the home setting in addition to taking care of patients on the nursing ward. Nearly all nurses (15/16, 94%) think that continuous monitoring in the home setting involves risks including a lack of the clinical view, occurrence of complications in the home setting, when complications remain unnoticed (for too long) and technical issues (e.g., Wi-Fi connection or defective sensor). Nurses also perceive using the sensor for patients with low health literacy or poor coping mechanisms as a risk.

*“There are definitely risks in the home setting. There must always be somebody who can take action if a patient calls or when you receive an alarm with the measurements of this patient. These are the measurements of this patient, who is responsible for taking action? There are quite a number of challenges [regarding monitoring in the home setting].”*

#### Relative priority

##### Experience on the nursing ward

This is defined as the degree to which nurses perceived continuous monitoring to be a priority in the organization and their department. Although the responses varied, most nurses (11/16, 69%) thought that the implementation of continuous monitoring would be a priority for the hospital. However, the priority on the nursing ward itself varied during implementation; 19% (3/16) considered it a priority during implementation, 19% (3/16, 19%) thought it was not a priority. All three hospitals conducted a pilot; 19% (3/16, 19%) mentioned that the priority decreased due to the unsuccessful pilot, and that there was a lack of priority (6/16, 38%) on the nursing ward after the pilot had ended.

*“I think priority is high, because a lot of manpower and money is dedicated to it.”*

#### Goals and feedback

##### Experience on the nursing ward

All respondents (16/16, 100%) could explain the aim of the intervention i.e. early detection of deterioration and the prospect of early discharge with continuous monitoring in the home setting.

*“Eventually, the goal is to discharge a patient early and to monitor them at home”*

#### Learning climate

##### Experience on the nursing ward

Learning climate refers to the degree to which nurses feel it was possible to give input, whether their input was valued, sufficient opportunity was given to try out the new intervention, sufficient time was available for learning, and how they felt about making mistakes. It was possible to give input (9/16, 56%) and the input was valued (12/16, 75%). Almost all nurses (12/16, 75%) had enough time for training. However, their perceptions about the possibility to test the intervention and whether they felt safe to try the intervention and make mistakes varied.

*“It was a pilot and it was no direct risk for the patient. We also performed the normal checks, so you had a good view of the patient and patient safety was not at risk”.*

#### Available resources

##### Experience on the nursing ward

This domain refers to the available resources and time for implementation. Nurses’ experiences varied, 81% (13/16) thought there were sufficient additional resources such as a dedicated project team and technical support. The majority (11/16, 69%) did not receive extra time for the intervention and 37% (6/16) reported that there were not enough human resources available during implementation. In particular, there was a lack of dedicated nurses.

*“There was a project team with supervisors and researchers and somebody from the technical department.”*

##### Expectations for continuous monitoring in the home setting

In total, 38% (6/16) thought that the current staffing is insufficient to handle the additional tasks for continuous monitoring in the home setting, and that extra human resources (4/16, 25%) would be beneficial for implementation.

*“If you also have patients here, you don’t have time for the patients at home. You need an extra person per shift, responsible for monitoring [in the home setting]”*

#### Access to information and knowledge

##### Experiences on the nursing ward

Access to information and knowledge included access to a manual and training on how to execute tasks associated with the intervention. Overall, this was rated positively by almost all nurses (15/16, 94%), especially a manual was perceived to be helpful. In total, 63% (10/16) was positive about the training. However, four nurses (4/16, 25%) were less satisfied, reasons being that a lot of information was given at once and they felt that there was insufficient opportunity to practice during the training.

*“The manual was changed frequently, with new tips and things. That was very useful!”*

##### Expectations for continuous monitoring in the home setting

In total, 75% (12/16) of the nurses think that information, for example a decision tree, or training would be beneficial for continuous monitoring in the home setting.

*“I think we need a manual on what to do with which complaints. It needs to be unequivocal.”*

### Characteristics of individuals

#### Knowledge and beliefs

##### Experiences on the nursing ward

This domain included statements on nurses’ beliefs about and attitudes towards continuous monitoring. Nurses were predominantly positive (7/16, 44%) about continuous monitoring on the nursing ward. In total, 25% (4/16) was not positive about the intervention.

*“I think it is a very nice development. When I see it in practice, I think it could be possible…there are a lot of patients that could just go home.”*

##### Expectations for continuous monitoring in the home setting

Nurses’ beliefs regarding continuous monitoring in the home setting varied; 56% (9/16) was positive about continuous monitoring in the home setting and they think it is a positive development, though 38% (6/16) was less enthusiastic about the development, especially because of the change in providing care.

*“I think this is a logical development in the sense that you always keep considering how care can be organized differently; you evolve with the time, technology develops rapidly, and I can understand that you start thinking about how you can monitor people at home, does that result in early discharge, and what can be done safely.”*

#### Other personal attributes

##### Experience from use on the nursing ward

This domain includes personal characteristics affecting implementation such as competence, age, employment, and experience with the intervention. In total, 75% (12/16) mentioned personal characteristics that will contribute to the implementation, for example (younger) age (2/16, 13%). Also, according to 63% (10/16) of nurses, experience with the new intervention tasks will be beneficial, for example to execute tasks correctly and at a more rapid pace.

*“The more often you do it, the easier it will become and you will get into a routine.”*

### Process

#### Formally appointed internal implementation leaders

##### Experience from use on the nursing ward

Six nurses from all three hospitals mentioned that a formally appointed internal implementation leader, often a project leader, was appointed to coordinate the intervention project. This was seen as positive by 44% (7/16) of the nurses because of the support and motivation they received.

*“The project leader was accessible, and visible on the nursing ward….I think that is important especially at the start, that somebody is always available to answer your questions”*

#### Champions

##### Experience from use on the nursing ward

Champions were mostly referred to as “key users”, a group of nurses with specific involvement and focus on this project. Champions were reported to be present in all three hospitals and their presence was appreciated by more than half (10/16, 63%) of all nurses, for example for practical support.

*“We had key-users who helped us attaching and connecting the sensor.”*

#### Reflecting and evaluation

##### Experience from use on the nursing ward

Over half of the nurses (9/16, 56%) were positive about the evaluation of the intervention’s implementation. They reported that evaluations, conducted during or after the implementation period, were completed in (team) meetings or that evaluation forms were used. This provided them with insights into the status of the implementation project. Almost 40% (6/16) was not involved in an evaluation or would have preferred an evaluation.

*“We discussed it each day in the daily evaluation. How is it going, is the connection working, are the check-ups good, do you notice differences, do you feel positively or negatively about it. A lot of attention was paid to it.”*

#### Facilitating conditions (UTAUT)

##### Experience from use on the nursing ward

Facilitating conditions concern the extent to which nurses perceive that technical infrastructure is adequate to support the intervention. This was considered negatively by half of the nurses (8/16, 50%). This was mainly due to a bad Wi-Fi connection (7/16, 44%), which was the main reason for discontinuing the pilot in two hospitals. Lack of interoperability with already existing systems, for example with the Electronic Medical Record (EMR), was also seen as a negative aspect by 25% (4/16).

*“The Wi-Fi was a problem. Sometimes the sensor did not connect and we had to restart the whole system. So that was the reason it did not work out.”*

### Suggestions and technical conditions for further development of continuous monitoring on the nursing ward and in the home setting

#### Suggestions for further development

In total, 12 nurses (12/16, 75%) provided suggestions for further development of continuous monitoring in the hospital and the home setting. Seven nurses (7/16, 44%) mentioned that they need additional parameters for continuous monitoring inside and outside the hospital, for example blood pressure or oxygen saturation. Other suggestions for improvement of continuous monitoring in the home setting include: agreements upon responsibilities for continuous monitoring in the home setting (3/16, 19%), personalized target values of vital parameters to prevent false alarms (2/16, 13%), and a dedicated contact person (2/16, 13%).

#### Conditions for continuous monitoring

To ensure successful intervention, interoperability with already existing systems (e.g. EMR) is perceived as important by nurses (8/16, 50%). This could contribute to (future) implementation and reduce nurses’ workload at the same time, by eliminating the need to manually register the sensor measurements in the EMR. Other conditions for continuous monitoring include properly working and reliable technology (network, sensor, etc.) (6/16, 38%), which will also lead to (extra) added value of this intervention. In addition, patients’ home situation should be ready (1/16, 6%), and patients should have the necessary skills (2/16, 13%) to use the sensor, before continuous monitoring can be implemented at home.

## Discussion

### Principal findings

In total, we identified 27 constructs from the CFIR framework and 1 construct from the UTAUT model influencing implementation of continuous monitoring on nursing wards. Five constructs that were experienced as a strong positive influence on implementation were mentioned by at least 8 nurses. These included relative advantage (e.g. early detection of deterioration), patient needs and resources (e.g. feeling safe), networks and communications (e.g. execute tasks together), personal attributes (e.g. experience with intervention), and implementation leaders (e.g. project leader). Five constructs were associated with a strong negative influence on implementation, including evidence strength and quality (e.g. lack of evidence from practical experience), complexity (e.g. number of procedural steps), design quality and packaging (e.g. bad sensor quality), compatibility (e.g. change in work) and facilitating conditions (e.g. Wi-Fi connection). Nurses expected continuous monitoring in the home setting to be hindered by compatibility with work processes and systems (e.g. change in work) and evidence strength and quality (e.g. lack of available evidence), and to be facilitated by access to knowledge and information (e.g. training) and perceived advantages of the implementation (e.g. data availability). Technical facilitating conditions, such as interoperability with existing systems, were suggested to be beneficial for further development.

### Comparison with other studies

Only a limited number of earlier studies evaluated nurses’ perspectives of continuous monitoring with wireless wearable devices [3, 9]. In a randomized controlled trial (RCT), health care professionals’ experiences with and expectations for use of a wearable device on a general ward were assessed using interviews. Several findings from this study were comparable with our study. They also found positive aspects to include early detection of clinical deterioration, feelings of safety, and shorter hospital stay. Negative aspects that were similar included less patient contact and not being able to measure all vital signs with one sensor. However, the results of this RCT also indicate that continuous monitoring can have both positive and negative effects on workload and time spent [8]. The findings of an observational cohort study on continuous monitoring with a wearable device on a general ward described that the majority of nurses (74%, *n* = 17) did not think that using the wearable device would be time-saving [24].

Continuous monitoring was perceived as complex especially due to extra time required for the intervention and the number of procedural steps to activate the sensor, such as attaching and connecting the sensor. This experience could also be a result of the pilot study setting, since this setting led to temporary duplications in registration and additional tasks, as multiple systems were used. Nurses also reported that current staffing was insufficient to monitor patients on the nursing ward and in the home setting simultaneously, and that additional (human) resources are necessary for the use of continuous monitoring in the home setting.

Integration of and compatibility with work processes and changed roles for professionals are found to be important for implementation of interventions using information and communication technology [25], such as continuous monitoring. We found that nurses’ lack of direct observation of the patient and the resulting inability to rely on their “clinical view” was perceived as a (possible) risk associated with continuous monitoring. They also expected that its use in the home setting will have a negative effect on their contact with patients, for example because of early discharge resulting in a shorter period of time during which nurses are able to interact with patients. Nurses in several previous studies were also worried about a decrease in patient contact [8, 15] making it more difficult to recognize deterioration [15]. The use of technology may change nurses’ profession and their contact and relationship with patients, especially in the case of remote care and monitoring. According to Peplau’s theory of interpersonal relations, contact between patients and nurses consists of different phases: orientation, identification, exploitation, and resolution. During these phases nurses can take on different roles, such as counsellor, technical expert, and resource person, for example to provide information [26]. The introduction of technology, such as sensor devices, may change the delivery of care, for example because patients are monitored remotely from home. This may also require a change in nurses’ roles because physical and face-to-face contact is more limited.

The success of an intervention is obviously affected by technology aspects and integration with current systems, including the hospital information technology infrastructure. This includes interoperability with the EMR [1], which is important for long-term use [1]. Additionally, a highly reliable Wi-Fi network is crucial for the success of continuous monitoring, as a number of nurses stated that the intervention was discontinued because the Wi-Fi network was unreliable. It was also found to be a barrier in a previous pilot study of continuous monitoring on a nursing ward [27]. Wi-Fi related issues can also cause data loss [28]. Therefore, prior to the implementation of wireless wearable sensors, it is recommended to ensure a well-functioning and reliable hospital Wi-Fi infrastructure [1]. Other technical issues included lack of evidence for the use of continuous monitoring as nurses sometimes experienced deviating measurements in comparison with another monitoring device used in daily practice. Evaluation of validity and feasibility of these devices is still ongoing [9]. This is especially relevant since the development of technology is evolving at a rapid pace, and currently multiple sensors, with different specifications, are available for continuous monitoring [9]. Therefore, pragmatic evaluation of new technologies, or new versions of existing technologies, is required.

Nurses’ personal characteristics may also affect the uptake of technology in clinical practice. eHealth literacy, “the ability to seek, find, understand, and appraise health information from electronic sources and apply the knowledge gained to addressing or solving a health problem” requires skills [29] and access to digital tools [30]. Nurses’ digital competencies can be affected by age and experience and may be improved by training and education [31]. Nurses in our study also highlighted that information (e.g., manual and decision tree) and training is needed, especially for continuous monitoring in the home setting. Also, technical support can facilitate technology use [32]. Another important aspect for successful digital interventions is technology acceptance. The UTAUT model can be used to assess both the intention for technology use and the actual use [18] and includes potential moderating factors such as age, experience, sex, and voluntariness of use. Other personal characteristics may also influence eHealth acceptance such as work experience, as well as knowledge about and experience with IT [33]. In future research, additional attention should be paid to the impact of nurses’ eHealth literacy, digital skills, and acceptance of interventions supported by technology.

Although several studies evaluated the perspectives and experiences of continuous monitoring from nurses’ perspectives [8, 15, 24, 27], there is limited information available about factors that influence implementation on different general wards and expectations for use of wireless wearable sensors in the home setting. Our overview, therefore, adds to the current body of knowledge by structured application of both CFIR and UTAUT frameworks. Future research is needed to confirm the use of this overview in developing, implementing, and evaluating interventions on a larger scale.

### Strengths and limitations

One strength of our study is that a wide range of factors was structurally assessed with focus on both experience from use of continuous monitoring on nursing wards and expectations of its use in the home setting. Additionally, we interviewed nurses from different teaching hospitals in which continuous monitoring was used in different populations and received comparable views on the use of the sensor. As indicated in the methods, not all constructs of the CFIR framework were used for the semi-structured interviews, but the included constructs were based on a selection made by the authors taking into consideration the type of intervention (continuous monitoring), the setting (hospital) and the respondents (nurses). However, topics related to other constructs (e.g., trialability, patient needs & resources and other personal attributes) came up during the interviews and were coded as belonging to these topics. This study has some limitations. Ten interviews were conducted face-to-face, while 6 interviews had to be conducted by telephone, due to COVID-19 circumstances. We do not think that this influenced the results, because a semi-structured interview was used and no additional notes were taken into account for data analysis, for example about non-verbal behavior. The first author conducted all interviews, and the transcripts were anonymized. Data analysis was conducted by the first two authors independently, of which one was not involved in the interviews. The preunderstanding of the authors was not used in the analysis. Furthermore, the number of nurses per hospital varied and continuous monitoring using wireless wearable sensors was conducted on different nursing wards in each hospital. Also, nurses’ personal characteristics (e.g., age, work experience and experience with the intervention) may have differed. Because saturation was confirmed (post hoc), we believe that all factors influencing implementation in this setting have been identified. The sample size was insufficient to look into differences between answers given by nurses with different characteristics. Future research is needed on the effect of nurses’ personal characteristics such as age, work experience, and (digital) skills on implementation of digital interventions such as continuous monitoring. Despite these limitations, this is to the best of our knowledge, the first qualitative study to identify and score constructs influencing the implementation of continuous monitoring on nursing wards and to classify perceptions on its use in the home setting.

## Conclusions

This paper provides an overview of factors influencing the implementation of continuous monitoring on nursing wards, including their relative importance, and provides insight in nurses’ perception of factors affecting its use in the home setting. This may be used as guidance for future implementations and evaluations.

## Supplementary Information

**Additional file 1.** Interview guide.

**Additional file 2.** Criteria used to assign ratings.

**Additional file 3.** Memo template (example).

**Additional file 4.** COREQ checklist.

**Additional file 5.** Continuous monitoring on the nursing ward: ratings assigned to CFIR and UTAUT constructs.

**Additional file 6.** Continuous monitoring in the home setting: ratings assigned to CFIR and UTAUT constructs.

## Data Availability

The datasets used and/or analyzed during the current study are available from the corresponding author on reasonable request.

## Abbreviations

CFIR: Consolidated Framework for Implementation Research
EMR: Electronic Medical Record
MEWS: Modified Early Warning Score
UTAUT: Unified Theory of Acceptance and Use of Technology
