# Supplementary material for: Remote continuous monitoring with wireless wearable sensors in clinical practice, nurses perspectives on factors affecting implementation: a qualitative study
Source: BMC Nurs. 2022 Mar 7;21:53. doi: 10.1186/s12912-022-00832-2 (PMC8899789; doi:10.1186/s12912-022-00832-2)
Supplement: Supplementary file 1 — Additional file 1. Interview guide. [file 12912_2022_832_MOESM1_ESM.docx]

# Additional file 1. Interview guide and CFIR constructs

Table 1. Interview guide

| **Domains** | **Questions** |
| --- | --- |
| 1. Intervention source^a^ | −^b^ |
| 1. Evidence Strength & Quality | - Do you think there is enough evidence that the sensor will work in the home setting? |
| 1. Relative advantage | - According to you, what is the advantage of using the sensor on the nursing ward? And in the home setting? |
|  | - Do you think there are other ways to achieve this goal (continuous monitoring in the home setting)? |
|  | - What are, according to you, the (dis)advantages for patients on the nursing ward/in the home setting?^b^ |
| 1. Adaptability^c^ | - In what way could the sensor be adapted to support your work? |
| 1. Trialability^d^ | − |
| 1. Complexity | - Hospital 1: Rate every task on a scale from 1: difficult to 10: easy, and explain this by mentioning barriers and facilitators? Were you able to execute this task alone? |
|  | - Hospital 2 and 3: Which tasks did you execute to enable monitoring with the sensor? On a scale from 1: difficult to 10:easy, how difficult were these tasks? - Hospital 2 and 3: Were there any barriers or facilitators? If yes, which?^e^ - Were you able to execute the tasks alone or were you dependent on others? |
|  | - Is it taken into account that extra time is needed for all these tasks? In other words, did you receive extra time for these tasks? |
|  | - One a scale from 1: difficult to 10: easy, how easy do you think it is to deliver care for patients using the sensor in the home setting? Do you think you need additional training, skills or information/knowledge? |
| 1. Design Quality and Packaging | - What is your opinion on the quality of the sensor? |
| 1. Cost^a^ | − |
| 1. Patient Needs & Resources^d^ | − |
| 1. Cosmopolitanism | - How do you think this care should be organized in the home setting?^f^ |
| 1. Peer Pressure^a^ | − |
| 1. External Policy & Incentive^a^ | − |
| 1. Structural Characteristics^a^ | − |
| 1. Networks & Communications | - What helped you the most: information via the project organization or supervisor in planned meetings, or unplanned information, for example during a coffee break or with a colleague? |
| 1. Culture^d^ | − |
| 1. Implementation Climate |  |
| 1. Tension for change | - Do you think the current monitoring method (*e.g. MEWS*) should be changed? - How can you see the (deviating) values? How are patients monitored in the current situation?^f^ |
| 1. Compatibility | - Do you think your relation with patients will change with continuous monitoring using the sensor? |
|  | - How is continuous monitoring going to help you with your work? Do you think you can do your job better? |
|  | - Do you think continuous monitoring will change your work on the nursing ward/in the home setting? |
|  | - Do you think there are risks for using the sensor in the home setting? |
| 1. Relative priority | - Is the use of the sensor a high priority in the hospital? |
|  | - And at the nursing ward? |
| 1. Organizational Incentives & Rewards^a^ | - |
| 1. Goals and Feedback | - Did you hear, in advance, what the aim is of using the sensor? What is the aim according to you? |
| 1. Learning Climate | - Was sufficient input asked from you? By whom? And was your input valued? |
|  | - Do you think there were enough possibilities to test (‘try-out’) the sensor? |
|  | - Was there enough time (training)? |
|  | - Were you worried about making mistakes? |
| Readiness for Implementation |  |
| 1. Leadership Engagement | − |
| 1. Available resources | - Do you think the hospital has enough resources (technical and human resources) available to support the use of the sensor? |
| 1. Access to knowledge and information | - What did you think of the training? - Do you think you need training, additional knowledge or skills?^e^ |
| 1. Knowledge & Beliefs | - The aim is to use continuous monitoring for patients in the home setting. What do you think about that? |
| 1. Self-efficacy | - Did you feel confident enough to communicate about this (continuous monitoring) with patients?^f^ |
| 1. Individual Stage of Change^d^ | − |
| 1. Individual Identification with Organization^d^ | − |
| 1. Other personal attributes^d^ | − |
| 1. Planning^d^ | − |
| 1. Engaging^a^ | − |
| 1. Opinion Leaders | - Were you motivated by people (in the hospital) to use the sensor? If yes, by whom? |
| 1. Formally Appointed Internal Implementation Leaders | - Were there people coordinating the project – the use of the sensor? - Was this beneficial? If no, did you miss something? |
| 1. Champions^d^ | − |
| 1. External Change Agents^d^ | − |
| 1. Executing^d^ | − |
| 1. Reflecting & Evaluating | - Was the project evaluated with you? What did you think about this? |

^a^ no results available

^b^ −: no question formulated

^c^ answers to questions were coded in different factors

^d^ results are available for this factor

^e^ question was missing in 1 interview

^f^ question was not posed to all respondents
