# Supplementary material for: Remote continuous monitoring with wireless wearable sensors in clinical practice, nurses perspectives on factors affecting implementation: a qualitative study
Source: BMC Nurs. 2022 Mar 7;21:53. doi: 10.1186/s12912-022-00832-2 (PMC8899789; doi:10.1186/s12912-022-00832-2)
Supplement: Supplementary file 2 — Additional file 2. Criteria used to assign ratings. [file 12912_2022_832_MOESM2_ESM.docx]

**Additional file 2: Criteria used to assign ratings**

Table 1. Criteria used to assign ratings to quotes [21]

| Rating | Criteria |
| --- | --- |
| -2 | The construct is a negative influence in the organization, an impeding influence in work processes, and/or an impeding influence in  implementation efforts. The majority of interviewees (at least two) describe explicit examples of how the key or all aspects (or the absence)  of a construct manifests itself in a negative way. |
| -1 | The construct is a negative influence in the organization, an impeding influence in work processes, and/or an impeding influence in  implementation efforts. Interviewees make general statements about the construct manifesting in a negative way but without concrete  examples:  • The construct is mentioned only in passing or at a high level without examples or evidence of actual, concrete descriptions of how that  construct manifests;  • There is a mixed effect of different aspects of the construct but with a general overall negative effect;  • There is sufficient information to make an indirect inference about the generally negative influence; and/or  • Judged as weakly negative by the absence of the construct. |
| 0 | A construct has neutral influence if:  • It appears to have neutral effect (purely descriptive) or is only mentioned generically without valence;  • There is no evidence of positive or negative influence;  • Credible or reliable interviewees contradict each other  • There are positive and negative influences at different levels in the organization that balance each other out; and/or different aspects of  the construct have positive influence while others have negative influence and overall, the effect is neutral. |
| +1 | The construct is a positive influence in the organization, a facilitating influence in work processes, and/or a facilitating influence in  implementation efforts. Interviewees make general statements about the construct manifesting in a positive way but without concrete  examples:  • The construct is mentioned only in passing or at a high level without examples or evidence of actual, concrete descriptions of how that  construct manifests;  • There is a mixed effect of different aspects of the construct but with a general overall positive effect; and/or  • There is sufficient information to make an indirect inference about the generally positive influence. |
| +2 | The construct is a positive influence in the organization, a facilitating influence in work processes, and/or a facilitating influence in  implementation efforts. The majority of interviewees (at least two) describe explicit examples of how the key or all aspects of a construct  manifests itself in a positive way.  Missing Interviewee(s) were not asked about the presence or influence of the construct; or if asked about a construct, their responses did  not correspond to the intended construct and were instead coded to another construct. Interviewee(s) lack of knowledge about a construct  does not necessarily indicate missing data and may instead indicate the absence of the construct. |
