# Supplementary material for: Remote continuous monitoring with wireless wearable sensors in clinical practice, nurses perspectives on factors affecting implementation: a qualitative study
Source: BMC Nurs. 2022 Mar 7;21:53. doi: 10.1186/s12912-022-00832-2 (PMC8899789; doi:10.1186/s12912-022-00832-2)
Supplement: Supplementary file 3 — Additional file 3. Memo template (example). [file 12912_2022_832_MOESM3_ESM.docx]

## Additional file 3. Memo template example

### C. Relative Advantage

***RATING: OVERALL +2 (ANALYST ONE +2, ANALYST TWO +2)***

***RATING – Continuous monitoring in the home setting: OVERALL +2 (ANALYST ONE +2, ANALYST TWO +2)***

*SUMMARY:* Relative advantage was (mainly) a positive construct and multiple example were mentioned.

*RATIONALE:* Relative advantage for continuous monitoring at the nursing ward included.

*DATA:*

Respondent 1:

Quote 1:33: *“And for patients ofcourse, because the patient is also, I think it is safer because we don’t do check-ups everytime here”*

- Valence and strenght: positive, +2
- Subcategory: Patient safety

Quote 1:35: “*If there is risk for the patient, that you notice that or at least receive an alarm, so I think you will get there earlier. Could be.*”

- Valence and strength: positive, +2
- Subcategory: Early deterioration

Quote 1:36. *“I think it is easier, it will take less time to do the same check-ups”.*

- Valence and strenght: Positive, +2
- Subcategory: Time/efficiency

Etc…

#### Continuous monitoring on the nursing ward

Table C1. Total number of quotes and rating per respondent

| **Respondents** | **Total No. quotes** | **Rating - No. quotes** | | | | |
| --- | --- | --- | --- | --- | --- | --- |
|  |  | -2 | -1 | 0 | 1 | 2 |
| 1 | 7 | - | - | - | - | 7 |
| 2 | 5 | - | - | - | 1 | 4 |
| 3 | 10 | - | - | 2 | 3 | 5 |
| 4 | - | - | - | - | - | - |
| 5 | 2 | - | - | - | - | 2 |
| 6 | 3 | - | - | 1 | 1 | 1 |
| 7 | 1 | - | 1 | - | - | - |
| 8 | 3 | - | - | - | - | 3 |
| 9 | 2 | - | - | - | - | 2 |
| 10 | 3 | - | - | - | - | 3 |
| 11 | 1 | - | - | - | 1 | - |
| 12 | 4 | - | - | - | - | 4 |
| 13 | 4 | - | - | - | 1 | 3 |
| 14 | 5 | - | - | - | 1 | 4 |
| 15 | 8 | 2 | - | - | - | 6 |
| 16 | 3 | - | - | - | 2 | 1 |
| Total | 61 (n=15) | 2 (n=1) | 1 (n=1) | 3 (n=2) | 10 (n=7) | 45 (n=13) |

Table C2. Ratings (neg/neutral/pos) per subcategory

| Categories | No. respondents (no. quotes) | |  |  |
| --- | --- | --- | --- | --- |
|  | Total | Negative  (-1 or -2) | Neutral  (0) | Positive  (+1 or +2) |
| Early deteriorating | 12 (22) | - | - | 12 (22) |
| Time and efficiency | 11(21) | 1(2) | 2(3) | 10(16) |
| Continuous monitoring – data availability | 7(7) | 1(1) | - | 6(6) |
| Patient safety | 4(7) | - | - | 4(7) |
| Quality (measurement/support clinical view) | 2(2) | - | - | 2(2) |
| Early discharge and (higher) turnover | 1(1) | - | - | 1(1) |

#### Continuous monitoring in the home setting

Table C4. Total number of quotes and rating per respondent (continuous monitoring in the home setting)

| **Respondents** | **Total No. quotes** | **Rating - No. quotes** | | | | |
| --- | --- | --- | --- | --- | --- | --- |
|  |  | -2 | -1 | 0 | 1 | 2 |
| 1 | - | - | - | - | - | - |
| 2 | 1 | - | - | - | - | 1 |
| 3 | 1 | - | - | - | - | 1 |
| 4 | - | - | - | - | - | - |
| 5 | 1 | - | - | - | - | 1 |
| 6 | 1 | - | - | - | - | 1 |
| 7 | 1 | - | - | 1 | - | - |
| 8 | 4 | - | - | - | - | 4 |
| 9 | - | - | - | - | - | - |
| 10 | 2 | - | - | - | 2 | - |
| 11 | - | - | - | - | - | - |
| 12 | 2 | - | - | 1 | 1 | - |
| 13 | 1 | - | - | - | - | 1 |
| 14 | 2 | - | - | - | - | 2 |
| 15 | - | - | - | - | - | - |
| 16 | - | - | - | - | - | - |
| Total | 16 (n=10) | - | - | 2 | 3 | 11 |

Table C5. Ratings (neg/neutral/pos) per subcategory

| Category | No. respondents (no. quotes) | |  |  |
| --- | --- | --- | --- | --- |
|  | Total | Negative  (-1 or -2) | Neutral  (0) | Positive  (+1 or +2) |
| Continuous monitoring – data availability | 4(4) | - | - | 4(4) |
| Early discharge and cost benefits | 3(3) | - | - | 3(3) |
| Early discharge and (higher) turnover | 3(4) | - | - | 3(4) |
| Early deteriorating | 2(2) | - | - | 2(2) |
| Time and efficiency | 2(2) | - | 2(2) | - |
| Patient safety | 1(1) | - | - | 1(1) |
