# Supplementary material for: Remote continuous monitoring with wireless wearable sensors in clinical practice, nurses perspectives on factors affecting implementation: a qualitative study
Source: BMC Nurs. 2022 Mar 7;21:53. doi: 10.1186/s12912-022-00832-2 (PMC8899789; doi:10.1186/s12912-022-00832-2)
Supplement: Supplementary file 5 — Additional file 5. Continuous monitoring on the nursing ward: ratings assigned to CFIR and UTAUT constructs. [file 12912_2022_832_MOESM5_ESM.docx]

## Additional file 5. Continuous monitoring on the nursing ward: ratings assigned to CFIR and UTAUT constructs

Table 1. CFIR and UTAUT domains and ratings for continuous monitoring on the nursing ward

| CFIR domains | | | | | Total rating^a^ | Total  N (no. of quotes^b^) | Negative  (-1 or -2) | Neutral (0) | Positive (1 or 2) |
| --- | --- | --- | --- | --- | --- | --- | --- | --- | --- |
| **I.Intervention characteristics** | | | | |  |  |  |  |  |
| **Evidence Strength and Quality** | | | | | **-2** | **14(36)** | **14(31)** | **1(1)** | **3(4)** |
|  | Evidence from practical experience | | | |  | 14(26) | 13(21) | 1(1) | 3(4) |
|  | Available evidence for continuous monitoring | | | |  | 2(10) | 2(10) | −^c^ | − |
| **Relative advantage** | | | | | **+2** | **15(61)** | **2(3)** | **2(3)** | **14(55)** |
|  | Early detection of deterioration | | | |  | 12(22) | − | − | 12(22) |
|  | Time/efficiency | | | |  | 11(22) | 1(2) | 2(3) | 10(17) |
|  | Continuous monitoring (data availability) | | | |  | 7(7) | 1(1) | − | 6(6) |
|  | Patient safety | | | |  | 4(7) | − | − | 4(7) |
|  | Quality (measurements/support clinical view) | | | |  | 2(2) | − | − | 2(2) |
|  | Early discharge and (higher) turnover | | | |  | 1(1) | − | − | 1(1) |
| **Trialability: pilot setting** | | | | | **Mixed** | **8(15)** | **3(5)** | **5(6)** | **3(4)** |
| **Complexity** | | | | | **-2** | **16(100)** | **15(87)** | **1(1)** | **6(12)** |
|  | Duration | | | |  | 13(59) | 13(59) | − | − |
|  | Perceived difficulty (intricacy) | | | |  | 13(29) | 8(17) | 1(1) | 6(11) |
|  | Number of procedural steps | | | |  | 9(12) | 8(11) | − | 1(1) |
| **Design Quality and Packaging** | | | | | **-2** | **15(39)** | **14(32)** |  | **5(7)** |
|  | Quality sensor | | | |  | 14(29) | 13(22) | − | 5(7) |
|  | Data availability | | | |  | 3(6) | 3(6) | − | − |
|  | Quality system | | | |  | 3(4) | 3(4) | − | − |
| **II. Outer setting** | | | | | | | | | |
| **Patient needs & resources** | | | | | **+2** | **10(25)** | **3(5)** | − | **10(20)** |
|  | Patient comfort (sensor burden) | | | |  | 8(14) | 3(5) | − | 5(9) |
|  | Feeling safe | | | |  | 5(6) | − | − | 5(6) |
|  | Patient mobility | | | |  | 2(2) | − | − | 1(2) |
|  | Information for patients | | | |  | 2(2) | − | − | 2(2) |
|  | Patient – attitude towards intervention | | | |  | 1(1) | − | − | 1(1) |
| **III. Inner setting** | | | | | | | | | |
| **Networks and communication** | | | | | **+2** | **15(32)** | − | **1(1)** | **15(31)** |
|  | Execute task together | | | |  | 10(15) | − | − | 10(15) |
|  | Formal communication | | | |  | 9(9) | − | 1(1) | 8(8) |
|  | Informal communication | | | |  | 5(6) | − | − | 5(6) |
|  | Formal and informal communication is necessary | | | |  | 2(2) | − | − | 2(2) |
| **Tension for change**: need to change current situation | | | | | **+1** | **13(18)** | **8(13)** | − | **5(5)** |
| **Compatibility** | | | | | **-2** | **13(39)** | **13(26)** | **7(8)** | **4(5)** |
|  | Compatibility with work process | | | |  | 12(21) | 12(19) | 1(1) | 1(1) |
|  |  | | Sensor limitations | |  | 6(10) | 6(10) | − | − |
|  |  | | Workload | |  | 4(5) | 4(5) | − | − |
|  |  | | (false) alarms | |  | 4(5) | 3(3) | 1(1) | 1(1) |
|  |  | | Responsibility for tasks | |  | 1(1) | 1(1) | − | − |
|  | Change in work | | | |  | 10(11) | − | 7(7) | 3(4) |
|  |  | | Clinical view | |  | 4(4) | − | 4(4) | − |
|  |  | | Addition to (current) work | |  | 2(2) | − | − | 2(2) |
|  |  | | Use of technology | |  | 2(2) | − | − | 2(2) |
|  |  | | Contact with specialist | |  | 1(1) | − | 1(1) | − |
|  |  | | Change in tasks | |  | 2(2) | − | 2(2) | − |
|  | Risk | | | |  | 6(7) | 6(7) | − | − |
|  |  | | | Lack of clinical view |  | 4(5) | 4(5) | − | − |
|  |  | | | Technology |  | 2(2) | 2(2) | − | − |
| **Relative priority** | | | | | **Mixed** | **16(39)** | **11(17)** | **1(1)** | **14(21)** |
|  | Nurses/nursing ward | | | |  | 12(19) | 10(13) | 1(1) | 4(5) |
|  |  | Priority during implementation | | |  | 7(8) | 3(3) | 1(1) | 3(4) |
|  |  | Priority after implementation/pilot | | |  | 7(8) | 6(7) | − | 1(1) |
|  |  | Priority decreased | | |  | 3(3) | 3(3) | − | − |
|  | Hospital | | | |  | 14(18) | 3(4) | − | 11(14) |
|  | Specialist(s) | | | |  | 2(2) | − | − | 2(2) |
| **Goals and Feedback** | | | | | **+1** | **16(20)** | − | − | **16(20)** |
| **Learning Climate** | | | | | **+1** | **16(79)** | **10(20)** | − | **16(59)** |
|  | Feeling safe to try/making mistakes | | | |  | 14(23) | 6(8) | − | 11(15) |
|  | Time for training | | | |  | 13(16) | 1(1) | − | 12(15) |
|  | Input was valued | | | |  | 12(12) | − | − | 12(12) |
|  | Possible to test intervention | | | |  | 11(15) | 7(9) | − | 5(6) |
|  | Possible to give input | | | |  | 10(13) | 1(2) | − | 9(11) |
| **Leadership engagement** | | | | | **1** | **2(3)** | **2(2)** | **1(1)** | − |
| **Available resources** | | | | | **Mixed** | **16(42)** | **13(20)** | − | **13(22)** |
|  | Available human resources during implementation | | | |  | 16(31) | 6(9) | − | 13(22) |
|  | Extra time for intervention | | | |  | 11(11) | 11(11) | − | − |
| **Access to information and knowledge** | | | | | **+1** | **16(48)** | **4(6)** | **3(4)** | **15(38)** |
|  | Manual | | | |  | 10(21) | − | 1(1) | 9(20) |
|  | Training | | | |  | 15(27) | 4(6) | 3(3) | 10(18) |
| **IV. Individual characteristics** | | | | | | | | | |
| **Knowledge and beliefs:** attitude towards intervention | | | | | **Mixed** | **9(12)** | **4(5)** | − | **7(7)** |
| **Individual stage of change**: change in enthusiasm | | | | | **Mixed** | **3(3)** | **2(2)** | − | **1(1)** |
| **Individual Identification with Organization** | | | | | **+2** | **1(1)** | − | − | **1(1)** |
| **Other personal attributes** | | | | | **+2** | **12(19)** | **2(2)** | **1(1)** | **12(16)** |
|  | Experience with executing (new)task | | | |  | 10(14) | − | 1(1) | 10(13) |
|  | (Younger) age | | | |  | 2(2) | − | − | 2(2) |
|  | Part-time employment | | | |  | 2(2) | 2(2) | − | − |
|  | Competence (communication) | | | |  | 1(1) | − | − | 1(1) |
| **V.Process** | | | | |  |  |  |  |  |
| **Planning** | | | | | **-2** | **4(4)** | **4(4)** | − | − |
| **Engaging:** | | | | |  |  |  |  |  |
| **Opinion leaders – experts (medical professionals)** | | | | |  | **3(4)** | **−** | **−** | **3(4)** |
| **Formally Appointed Internal Implementation Leaders** | | | | | **+2** | **10(26)** | **−** | **6(7)** | **7(19)** |
| **Champions** | | | | | **+1** | **14(34)** | **2(2)** | **10(14)** | **10(18)** |
| **External Change Agents** | | | | |  | **3(4)** | **−** | **1(1)** | **3(3)** |
| **Reflecting and Evaluating** | | | | | **Mixed** | **15(33)** | **6(7)** | **4(5)** | **9(21)** |
| **UTAUT** | | | | |  |  |  |  |  |
| **Facilitating conditions** | | | | | **-2** | **8(31)** | **8(31)** | − | − |
|  | (Wi-Fi) Connection | | | |  | 7(26) | 7(26) | − | − |
|  | Interoperability | | | |  | 4(5) | 4(5) | − | − |

^a^ Minus sign (-) means a negative influence on implementation, positive sign (+) means positive influence on implementation, ‘mixed’ means both negative and positive influence on implementation

^b^ In total, 1068 quotes were selected of which 5 quotes were coded to two constructs

^c^ “−”: construct was not mentioned by nurses
