# Supplementary material for: Remote continuous monitoring with wireless wearable sensors in clinical practice, nurses perspectives on factors affecting implementation: a qualitative study
Source: BMC Nurs. 2022 Mar 7;21:53. doi: 10.1186/s12912-022-00832-2 (PMC8899789; doi:10.1186/s12912-022-00832-2)
Supplement: Supplementary file 6 — Additional file 6. Continuous monitoring in the home setting: ratings assigned to CFIR and UTAUT constructs. [file 12912_2022_832_MOESM6_ESM.docx]

**Additional file 6. Continuous monitoring in the home setting: ratings assigned to CFIR and UTAUT constructs**

Table 1. CFIR and UTAUT domains and ratings for continuous monitoring in the home setting

| CFIR domains | | | Total rating^a^ | Total  N (no. of quotes^b^) | Negative (-1 or -2) | Neutral (0) | Positive (+1 or +2) |
| --- | --- | --- | --- | --- | --- | --- | --- |
| **I.Intervention characteristics** | | |  |  |  |  |  |
| **Evidence Strength and Quality:** available evidence for continuous monitoring in the home setting | | | **-1** | **8(18)** | **8(18)** | **−^c^** | **−** |
| **Relative advantage** | | | **+2** | **10(16)** | **−** | **2(2)** | **9(14)** |
|  | Continuous monitoring (data availability/access) | |  | 4(4) | − | − | 4(4) |
|  | Early discharge: (higher) turnover | |  | 3(4) | − | − | 3(4) |
|  | Early discharge: cost | |  | 3(3) | − | − | 3(3) |
|  | Early deterioration | |  | 2(2) | − | − | 2(2) |
|  | Time/efficiency | |  | 2(2) | − | 2(2) | − |
|  | Patient safety | |  | 1(1) | − | − | 1(1) |
| **Complexity** | | | **-2** | **7(8)** | **7(8)** | **−** | **−** |
|  | Perceived difficulty (intricacy) | |  | 5(5) | 5(5) | − | − |
|  | Duration | |  | 2(2) | 2(2) | − | − |
|  | Number of procedural steps | |  | 1(1) | 1(1) | − | − |
| **II. Outer setting** | | |  |  |  |  |  |
| **Patient needs & resources** | | | **Mixed** | **15(44)** | **12(17)** | **1(1)** | **14(26)** |
|  | Patient feeling safe | |  | 13(19) | 10(13) | − | 5(6) |
|  | Recovery in own home | |  | 11(18) | − | 1(1) | 10(17) |
|  | Patient comfort/burden | |  | 3(4) | 3(4) | − | − |
|  | Information for patients | |  | 2(2) | − | − | 2(2) |
|  | Treatment adherence | |  | 1(1) | − | − | 1(1) |
| **Cosmopolitanism** | | | 1 | 3(6) | − | 2(2) | 2(4) |
| **III. Inner Setting** | | |  |  |  |  |  |
| **Culture:** change in culture | | | **0** | **2(2)** | **−** | **2(2)** | **−** |
| **Compatibility** | | | **-2** | **16(97)** | **16(72)** | **12(24)** | **1(1)** |
|  | Change in work | |  | 14(22) | 8(10) | 9(12) | − |
|  |  | Contact with patient |  | 13(16) | 7(9) | 7(7) | − |
|  |  | Change in tasks |  | 3(4) | − | 3(4) |  |
|  |  | Clinical view |  | 1(1) | 1(1) | − | − |
|  |  | Responsibility |  | 1(1) | − | 1(1) | − |
|  | Compatibility with work process | |  | 11(23) | 8(14) | 6(8) | 1(1) |
|  |  | Time/workload |  | 6(11) | 5(10) | 1(1) | − |
|  |  | Responsibility for tasks |  | 5(7) | 2(2) | 4(5) | − |
|  |  | (false) alarms |  | 2(2) | − | 2(2) | − |
|  |  | Applicability patient population |  | 2(2) | 1(1) | − | 1(1) |
|  |  | Sensor detachment |  | 1(1) | 1(1) | − | − |
|  | Risks | |  | 15(52) | 15(48) | 3(4) | − |
|  |  | Complications |  | 9(18) | 8(16) | 2(2) | − |
|  |  | Clinical view |  | 8(13) | 8(13) | − | − |
|  |  | Patient population: health skills/coping |  | 6(9) | 6(9) | − | − |
|  |  | Applicability to patient population |  | 4(5) | 4(4) | 1(1) | − |
|  |  | Technology |  | 3(3) | 2(2) | 1(1) | − |
|  |  | Responsibility |  | 3(3) | 3(3) | − | − |
|  |  | Sensor detachment: lack of data availability |  | 1(1) | 1(1) | − | − |
| **Available resources** | | | **Mixed** | **9(14)** | **6(8)** | **1(1)** | **4(5)** |
|  | Human resources available | |  | 6(8) | 6(8) | − | − |
|  | Human resources needed | |  | 5(6) | − | 1(1) | 4(5) |
| **Access to information and knowledge** Information (e.g. decision tree) or training is needed | | | **+2** | **13(21)** | **−** | **1(1)** | **12(20)** |
| **IV. Characteristics of individuals** | | |  |  |  |  |  |
| **Knowledge and beliefs:**  Attitude towards continuous monitoring in the home setting | | | **Mixed** | **12(26)** | **6(8)** | **1(1)** | **9(17)** |
| **Other personal attributes** | | | **+2** | **6(7)** | − | − | **6(7)** |
|  | Experience with executing (new) task | |  | 3(4) | − | − | 3(4) |
|  | Work experience | |  | 3(3) | − | − | 3(3) |

^a^ Minus sign (-) means a negative influence on implementation, positive sign (+) means positive influence on implementation, ‘mixed’ means both negative and positive influence on implementation

^b^ In total, 1068 quotes were selected of which 5 quotes were coded to two constructs

^c^ “−”: construct was not mentioned by nurses
